# ‘Mortgaged lives’: the biopolitics of debt and housing financialisation^1^

**DOI:** 10.1111/tran.12126

**Published:** 2016-06-01

**Authors:** Melissa García‐Lamarca, Maria Kaika

**Affiliations:** ^1^ GeographySchool of Environment, Education and DevelopmentUniversity of ManchesterManchesterM13 9PL

**Keywords:** real estate, financialisation, housing, Spain, mortgages, crisis

## Abstract

The paper expands the conceptual framework within which we examine mortgage debt by reconceptualising mortgages as a biotechnology: a technology of power over life that forges an intimate relationship between global financial markets, everyday life and human labour. Taking seriously the materiality of mortgage contracts as a means of forging new embodied practices of financialisation, we urge for the need to move beyond a policy‐ and macroeconomics‐based analysis of housing financialisation. We argue that more attention needs to be paid to how funnelling land‐related capital flows goes hand in hand with signing off significant parts of future labour, decisionmaking capacity and well‐being to mortgage debt repayments. The paper offers two key insights. First, it exemplifies how macroeconomic and policy changes could not have led to the financialisation of housing markets without a parallel biopolitical process that mobilised mortgage contracts to integrate the social reproduction of the workforce into speculative global real‐estate practices. Second, it expands the framework of analysis of emerging literature on financialisation and subjectification. Focusing on the mortgage defaults and evictions crisis in Spain, we document how during Spain's 1997–2007 real‐estate boom the promise of mortgages as a means to optimise income and wealth enrolled livelihoods into cycles of global financial and real‐estate speculation, as home security and future wealth became directly dependent on the fluctuations of financial products, interest rates and capital accumulation strategies rooted in the built environment. When, after 2008 unemployment escalated and housing prices collapsed, mortgages became a punitive technology that led to at least 500 000 foreclosures and over 250 000 evictions in Spain.

A debtor is a debtor for life. Even if they work twenty‐four hours a day they will never repay that debt. It is the perfect form of extortion and slavery. (Ada Colau, co‐founder and former spokesperson, Platform for Mortgage Affected People (PAH), Mayor of Barcelona since May 2015. Quoted in Muriel 2012, no page number)

## Introduction: the role of mortgages in governing life … or the biopolitics of financialising home ownership

This paper seeks to expand the conceptual framework and empirical basis within which we examine mortgage debt and housing financialisation. It does this by conceptualising mortgages as a biotechnology – a ‘technology of power over life’ (Foucault 2003, 246) – that engineers an increasingly intimate relationship between practices of everyday life and speculative practices of global real estate and financial markets. Taking seriously the materiality of mortgage contracts as a means of forging new embodied practices of financialisation, we urge for the need to move beyond a policy‐ and macroeconomic‐based analysis of housing financialisation and to pay more attention – empirically and conceptually – to how ‘channelling the flow of capital onto and through the land’ (Harvey 1982, 361) under a financialised global economy goes hand in hand with signing off significant parts of future labour, decisionmaking capacity and even health and well‐being to mortgage debt repayments. Through advancing Foucault's (1978, 141) proposition that capitalism needs not only productive bodies but also ‘the adjustment of the phenomena of population to economic processes’, we develop a conceptual framework and a qualitative, empirically grounded understanding of the link between the policies and macroeconomic processes that deregulate mortgage lending and the embodied practices of becoming indebted and defaulting on mortgage debt payments. By doing so, the paper contributes two key insights.

First, it challenges recent debates that seek to understand the mortgage crisis and the financialisation of housing primarily through macroeconomics and/or policy analysis (Aalbers 2008 2009 2011; Ashton 2012 Baldauf 2010; Christophers 2011 2015; Gotham 2009; Lapavitsas 2009; Wainwright 2009). The paper exemplifies how macroeconomic and policy changes could not have led to the financialisation of housing markets^2^ without a parallel biopolitical process that mobilised mortgage contracts to integrate the social reproduction of the workforce into speculative global real‐estate practices. We argue that mortgages are not just another form of ‘financial expropriation’, simply ‘an extraction of financial profit directly out of personal income’ (Lapavitsas 2009, 114). As we shall show, after the 1990s, the expansion of mortgage contracts and the promise of mortgaged home ownership as a means to optimise income and wealth entrenched not only the economic but also the social reproduction of the workforce into global cycles of financial speculation and led to an unprecedented expansion of urban capital accumulation (Fine 2010; Harvey 1978). In other words, mortgage contracts enrolled not only personal income, but also the practices of everyday life as well as community and family relations as cogwheels into the global speculative financial strategies that drive capitalist urbanisation. Drawing on ethnographic research in Spain, we show how countless people who signed mortgage contracts found their lives directly dependent on the success or failure of capital accumulation strategies rooted in the built environment (Rossi 2013). This occurred as they became embedded in a process where the production of surplus value is subsumed not only via the direct exploitation of their labour power, but also via the *circulation* of capital through a personalised debt relation that cannot be written‐off or broken, even after the asset (their home) is repossessed by the creditor (the bank). The embodied processes that enrolled millions of lives in global real‐estate speculation and debt servicing practices are, we argue, central in the macroeconomic process of ‘housing financialisation’.

Second, the paper offers new conceptual insight and empirical nuance to the emerging body of work on financialisation and subjectification (French and Kneale 2012; Hall 2011; Kear 2013; Langley 2006 2007 2008; Lazzarato 2012; Martin 2002) or financialisation as a ‘lived process’ (Kaika and Ruggiero 2013, 2015). In the pages that follow, mortgage contracts as tools for forging embodied practices of financialisation and for subjecting life to debt servicing practices are exemplified through the narratives of people who were ‘sold’ the idea of becoming leveraged real‐estate investors (Langley 2008), but were subsequently unable to service their mortgage debt. Some were even evicted from their mortgaged homes. The escalation of unemployment rates in Spain since 2008 has been the main driver behind the foreclosures of over 500 000 properties and the eviction of at least 250 000 families from their mortgaged homes. According to Spanish legislation, households still owe their mortgage debt plus interest and court fees even after they are evicted if the nominal value of the property at the moment of repossession is lower than the outstanding mortgage debt. Given the recent collapse in housing prices, this means that most households remain indebted, and people's lives remain subjected to debt servicing practices even after their mortgaged home is repossessed by banks. The stories of Spain's defaulting mortgage holders and evicted mortgaged ‘homeowners’ elucidate how mortgage contracts that were supposed to enhance social and economic status ended up affecting negatively not only people's housing and finances, but also their family and community relations, their personal health and a broad range of everyday practices in unexpected and unforeseen ways that span from decreased levels of self‐esteem and belonging, to health deterioration and increased levels of anxiety, guilt, stress and fear.

While mortgaged home ownership has always involved owing one's future labour to debt servicing practices, three significant developments intensified the role of mortgages as a biopolitical tool since the 1990s. First, the sheer scale of expansion of mortgage debt. This is illustrated by the fact that mortgages are the ‘single largest asset in people's everyday life’ (Schwartz and Seabrooke 2008, 237). Furthermore, as depicted in Table 1, by 2008, the ratio of residential mortgage debt to Gross Domestic Product (GDP) rose to over 250 per cent of its 1998 value in Spain, Italy and Ireland, over 500 per cent in Greece over 170 per cent in Finland, France, the Netherlands, Portugal and Sweden, and over 150 per cent in Belgium, Denmark and the UK. The Gross Debt to Income Ratio of households shows an even more striking change, with for example Spain and the Netherlands having doubled this ratio since 1995. Second, the astonishing expansion of mortgage credit availability was fuelled by an unprecedented expansion of practices of mortgage debt securitisation after the 1990s. This meant that household debt repayments and the security and (future) wealth of unprecedented numbers of households became directly dependent not only on the fluctuation of interest rates, but also on the performance of speculative global financial markets. Moreover, mortgage debt securitisation significantly changed the performance of mortgage contracts as biopolitics, as it mutated what used to be experienced as an embodied and often personalised debt relationship (usually with a clerk or director of a local bank branch) into a disembodied debt relationship with an opaque and unreachable global financial entity. As the narratives in this paper recount, the impossibility to put a name – let alone a face – on one's creditor after the securitisation of debt further accentuated mortgage defaults, practices of alienation and the entanglement of everyday life into the web of global financial markets. Third, and finally, in many countries the extension of mortgage credit availability went hand in glove not only with a speculative boom in housing prices, but also with increasing levels of labour precariousness. As evidenced in the soaring numbers of foreclosures and evictions in Spain (which tripled since 1995^3^), mortgaged subjects have never been more volatile. Spain's legislative framework combined with the massive subsequent fall in housing prices left people indebted even after their home was repossessed.

**Table 1:** Household debt characteristics of a selection of European countries

	1998	1999	2000	2001	2002	2003	2004	2005	2006	2007	2008	2009
*Belgium*												
Household mortgage debt to GDP (%)	26.5	27.6	27.7	26.7	27.8	30.1	30.7	33.4	35.9	37.7	39.8	43.3
Household debt to net disposable income (%)	68.3	70.5	68.3	66.3	68.3	70.7	74.6	79.5	83.3	87.4	89.8	90.8
*Denmark*												
Household mortgage debt to GDP (%)	67.5	68.6	67.7	71.1	74.0	78.4	79.7	84.9	89.1	93.1	95.4	103.8
Household debt to net disposable income (%)	221.1	226.3	232.8	237.7	242.9	248.7	261.9	282.1	299.4	324.7	339.4	338.7
*Finland*												
Household mortgage debt to GDP (%)	29.7	30.5	30.3	31.2	32.7	35.1	38.2	41.9	44.5	45.7	48.0	58.0
Household debt to net disposable income (%)	64.7	66.0	69.2	70.8	75.6	79.9	88.6	99.2	109.4	114.7	117.1	117.5
*France*												
Household mortgage debt to GDP (%)	20.0	20.8	21.2	21.7	22.6	24.2	26.0	29.2	32.0	34.4	36.4	38.0
Household debt to net disposable income (%)	72.4	74.9	74.8	77.0	77.5	81.1	81.9	88.4	93.6	96.6	98.7	104.3
*Greece*												
Household mortgage debt to GDP (%)	5.8	6.7	8.2	10.7	13.6	15.5	18.3	23.2	27.2	30.6	32.5	33.9
Household debt to net disposable income (%)	–	–	–	–	–	–	–	–	72.1	80.4	84.7	85.3
*Ireland*												
Household mortgage debt to GDP (%)	26.5	29.0	31.0	32.8	36.2	42.5	51.7	61.0	69.7	73.7	81.5	90.3
Household debt to net disposable income (%)				110.8	126.2	147.2	170.0	201.0	225.9	236.2	231.9	240.8
*Italy*												
Household mortgage debt to GDP (%)	7.8	9.0	9.8	9.9	11.0	13.0	14.8	17.0	18.6	19.7	19.6	21.7
Household debt to net disposable income (%)	46.0	50.7	54.5	56.5	59.4	62.5	66.2	71.3	76.1	80.2	81.6	86.5
*Netherlands*												
Household mortgage debt to GDP (%)	55.3	60.7	68.2	73.0	80.2	83.9	88.2	93.5	96.7	98.3	98.9	105.6
Household debt to net disposable income (%)	176.7	189.0	199.1	194.2	204.4	222.9	233.0	251.5	256.9	261.4	274.3	286.6
*Poland*												
Household mortgage debt to GDP (%)	1.5	1.7	2.1	2.7	3.4	4.5	4.7	6.0	8.4	11.6	15.6	18.2
Household debt to net disposable income (%)						19.7	21.6	25.0	31.2	39.2	51.5	52.8
*Portugal*												
Household mortgage debt to GDP (%)	n/a	36.9	41.5	44.4	47.9	47.8	49.3	53.3	59.1	62.0	63.2	67.5
Household debt to net disposable income (%)	77.0	94.3	106.8	118.3	121.6	123.6	126.8	135.9	140.6	145.7	148.9	151.4
*Spain*												
Household mortgage debt to GDP (%)	23.9	26.7	29.9	32.5	35.9	40.0	45.7	52.3	58.1	61.4	62.0	64.6
Household debt to net disposable income (%)		79.7	84.2	87.1	94.1	102.3	113.6	128.2	144.3	154.1	150.1	145.2
Number of mortgages granted	529 565	585 782	612 852	615 703	690 231	989 439	1 107 664	1 257 613	1 342 171	1 238 890	836 419	650 889
Amount of mortgages (billions of euros)	29.5	36.2	42.3	46.6	59	96.1	112.1	156.9	188.3	184.4	116.8	76.6
*Sweden*												
Household mortgage debt to GDP (%)	43.9	45.8	44.6	46.1	47.0	48.5	57.0	59.4	64.8	66.9	66.7	82.0
Household debt to net disposable income (%)	101.5	105.3	108.6	119.2	121.6	128.2	137.0	146.7	153.8	157.4	159.5	163.5
*UK*												
Household mortgage debt to GDP (%)	49.8	55.1	55.8	58.0	62.1	67.4	71.2	77.5	82.4	85.4	80.3	87.6
Household debt to net disposable income (%)	114.4	117.3	118.9	125.6	138.8	151.7	164.9	167.2	178.8	183.3	178.1	167.5

Although a longitudinal analysis of the biopolitical role of mortgages before the 1990s lies outside the scope of this paper, our intention here is to foreground these more recent processes that require systematic attention and detailed historical and geographical analysis. Apart from expanding the conceptual framework within which we analyse the making of biopolitical financial subjects and the socioeconomic consequences of mortgage debt, the paper also enhances the availability of qualitative data on Spain's recent mortgage crisis (Colau and Alemany 2012; Coq‐Huelva 2013; García 2010; López and Rodríguez 2010 2011; Naredo *et al*. 2007 2008; Palomera 2014; Puig Gómez 2011; Romero *et al*. 2012; Sánchez Martínez 2008). Our contribution draws on original material that includes 19 in‐depth interviews with mortgage‐affected people, 12 semi‐structured interviews with key informants from the policy and banking sector, one year of participant observation (from October 2013 to September 2014) at assemblies and actions of Barcelona's and Sabadell's Platform for Mortgage Affected People (PAH, Spain's grassroots anti‐eviction platform), archival data from the National Institute for Statistics (INE), the Spanish Tax Agency (AEAT), the Bank of Spain and the European Mortgage Federation (EMF).

## From financialising homes to financialising lives

The commodification of housing as a key engine of urban capital accumulation and as an ideal object of speculation is well documented in the academic literature (see, among others, Butler and Robson 2003; Fenton *et al*. 2013; Forrest and Williams 1984; Harvey 1978). While the use and exchange values of housing function in ways similar to any typical market commodity, housing has recently been promoted as an asset with the potential to act ‘as an investment … not only for the capitalist that produces it, but also for the homeowner that “consumes” it’ (Ivanova 2011, 862). Therefore, housing can affect the speculative aspects of the real‐estate market not only through its potential to stimulate investment and accumulation (Aalbers 2008; Harvey 1978), but also through its potential to affect the structure of consumption in general.

After the 1990s, significant institutional and macroeconomic changes at the national and international scale enabled the global expansion of speculative real‐estate investment through financial markets. Combined with the liberalisation of mortgage financing, these processes became central for an unprecedented expansion of mortgaged home ownership in many countries around the world (see Table 1) and gave impetus to what is now depicted by many scholars as the ‘financialisation of housing’ (Aalbers 2008, 2009, 2011; Christophers 2015; Rolnik 2013). As the liberalisation of mortgages was accompanied by a significant decline in welfare provision, the expansion of home ownership via mortgages contributed to the promotion of the idea of citizens not only as consumers but also as leveraged real‐estate investors (Case *et al*. 2005; Castles and Ferrera 1996; Ivanova 2011; Langley 2008; Toussaint and Elsinga 2009). The accumulation of housing wealth was promoted not only as a good investment, but also as a ‘buffer’ (Benito 2007), ‘cushion’ (Lowe *et al*. 2012), alternative ‘pension scheme’ (Aron *et al*. 2012; Dorling 2014; Forrest and Hirayama 2009; Malpass 2008; Powell 2008) and/or a private ‘asset‐based welfare’ system (Crouch 2009; Smith and Searle 2010) that could compensate for declining welfare provision (Desmond 2012; Finlayson 2009; Lowe *et al*. 2012; Powell 2008; Regan and Paxton 2001; Watson 2009).

However, the expansion of mortgage lending and the production of citizens as real‐estate investors accentuated not only speculative urban economic restructuring but inequality too (Bridge *et al*. 2014; Moulaert *et al*. 2003; Rutland 2010; Smart and Lee 2003; Weber 2002). It is now well documented that the macroeconomic and institutional changes that led to an intensification of the production of private housing as an ideal object of speculation (Butler and Robson 2003; Fenton *et al*. 2013; Smith and Searle 2010) were significant in accentuating the mortgage debt crisis (Aalbers 2008 2009 2011; Ashton 2012; Baldauf 2010; Christophers 2011 2015; Gotham 2009; Wainwright 2009) and intensifying social polarisation (Bridge *et al*. 2014; Lees *et al*. 2008; Rolnik 2013; Soederberg 2014; Wyly *et al*. 2009).

The body of research outlined above offered significant insight into the role that mortgages play internationally (by boosting the speculation of global financial markets and fuelling the recent economic crisis), nationally (by compensating for changes in welfare regimes) and locally (by being instrumental in forging urban socioeconomic change). However, its focus on macroeconomic changes and institutional and policy agendas has rarely documented or theorised the link between these changes and the embodied processes that enrol citizens in speculative global financial practices through mortgage contracts. This research, in other words, falls short of documenting the everyday practices of becoming indebted and living with mortgage debt defaults and evictions. Our contribution addresses this gap by examining how mortgages became the means to control and discipline ‘the random element inherent in a population of living beings’ (Foucault 2003, 246) and to secure capital circulation despite the contingency of life (Dillon 2007; Dillon and Lobo‐Guerrero 2008). We demonstrate how mortgaged home ownership became normalised as the smartest and most secure tenure option across the social spectrum. As long as real estate was booming, housing prices rose and employment was stable, it seemed to be a win–win model. But as inflation and interest rates crept up, mass unemployment hit and housing prices plummeted, the disciplinary and punitive dimension of mortgage contracts was revealed. As non‐performing mortgage debt became officially classified as junk, people holding this debt also became classified as ‘rubbish’ or ‘scum’, as one informant explains later in the paper.

As noted in the introduction, focusing on the embodied practices of housing financialisation, this paper also offers new conceptual and empirical insight to the emerging literature on financialisation and subjectification. This body of work unpacks how the liberalisation of the economy encourages citizens to change their subject positions and/or become strategic investors in order to meet the basic needs of everyday life – such as access to housing, healthcare, pensions – under a neoliberal economy (French and Kneale 2012; Hall 2011; Kaika and Ruggiero 2013 2015; Kear 2013; Langley 2006 2007 2008; Lazzarato 2012; Martin 2002). Langley's work (2006 2007 2008) is particularly pertinent to our analysis, as he documents the production of everyday investor identities (Langley 2007) in the context of Anglo‐American mortgage finance. Our exploration points to new dimensions of what French and Kneale (2012) term biofinancialisation, specifically how contemporary processes of financialisation intermesh and intertwine with the politics of life itself (Rose 2007), producing distinctive capital/life/subject relations.

In the sections that follow, we first briefly historicise the normalisation of home ownership in Spain during Franco's dictatorship (1939–1975). We next focus on how, during the decades that followed the end of Franco's rule (1975–1999), significant institutional and policy changes enabled mortgages to act as the key tool that was supposed to fulfil the promise for housing for all. We document how institutional and economic changes related to the liberalisation of mortgages triggered the largest real‐estate boom period in Spain's history (1999–2007). How the exponential increase in indebtedness of Spanish households was recorded statistically as an increase in home ownership‐related wealth, which was fictitious and based on speculatively inflated housing prices, is then detailed. The penultimate section documents the discourse and everyday practices that were central in convincing millions of households to sign up to mortgage contracts between 1999 and 2007. The final section centres on the post‐2007 period, and records how the economic crisis brought mortgage holders face to face with the violence of financial capitalism.

## ‘A country of homeowners, not proletarians’: inscribing home ownership into the DNA of Spaniards under Franco (1939–1975)

While 84 per cent of the Spanish population were registered as homeowners at the peak of the housing boom in 2006, home ownership had not always been a feasible or desirable option for Spaniards (Naredo 2004). At the aftermath of the Civil War (1936–1939), Spain was left with a housing stock of insufficient quantity and poor quality. Up until the 1950s, home ownership rates in Madrid, Barcelona, Seville and Bilbao were as low as five, six, ten and 12 per cent of these cities’ population respectively (Naredo 2010). But like in other southern European countries (see Allen *et al*. 2004), home ownership soon became an integral part in the process of state building. Franco's dictatorship, established in 1939, saw the provision of state‐subsidised housing as a means to enhance national stability and secure support for the regime, while promoting economic development (Cayuela Sánchez 2010). The first ever Spanish Minister of Housing (1957) was Falangist ideologue José Luis Arrese, who epitomised the role of housing in the regime's ideology in his famous statement ‘we want a country of homeowners, not proletarians’, and his promise to ‘make a spring of homes grow in Spain’ (Naredo 2010). Colau and Alemany (2012) note that the project to produce a nation of homeowners both sidestepped potential conflict between state and tenants and acted as a disciplinary mechanism that could convert potentially insubordinate spirits into orderly home‐owners/citizens. Within this spirit, the promotion of home ownership (particularly for low‐income households) as a socially admirable and ‘patriotic’ way of living evolved in parallel with an ideology that depicted renting and subleasing as socially unacceptable (Maestrojuan 1997, 183; see also Ronald 2008).

Existing literature offers detailed and critical accounts of Franco's housing policies (Leal 2005; López and Rodríguez 2010; Tatjer 2008). But it is important to note here that the Francoist period laid the foundations for radical changes in the practice and perception of the status of home ownership in Spain to such an extent that, as Colau and Alemany note, by the 1980s homeownership in Spain was considered completely natural, ‘like a lucky genetic code inscribed into our DNA that sets us apart from other mortals and determines our behaviour’ (2012, 33). By the end of Franco's dictatorship, over 60 per cent of Spaniards qualified as homeowners.

## Economic activity trumps social policy: instituting a nation of indebted homeowners (1975 onwards)

After the end of the dictatorship (1975), state provision of social housing dropped off policy agendas (López and Rodríguez 2010) and housing policy focused instead on stimulating private home ownership through policies that offered significant tax breaks for mortgage repayments on primary and secondary homes (Cabré and Módenes 2004). Up until the 1980s, mortgages were only available through the state‐controlled Spanish Public Mortgage Bank. However, the 1981 Mortgage Market Regulation Bill enabled private operators to enter the mortgage market. The Bill also increased the acceptable loan‐to‐value ratio for mortgages from 50 to 80 per cent, introduced variable interest rates and allowed banks to issue asset‐backed mortgage bonds (*cédulas hipotecarias*) as a means to expand mortgage financing (Alberdi 1997). A supplementary 1992 Bill also authorised the use of special purpose vehicles (SPVs)^4^ to securitise mortgages (López and Rodríguez 2010). This allowed Spanish banks to expand the financing of mortgages beyond the availability of their own bonds and deposits, as the demand for additional mortgage credit by far exceeded the growth in household savings (Banco de España 2007; Fuentes Egusquiza 2007; Martín‐Oliver and Saurina 2008).

Boosting home ownership through mortgages during the 1980s and 1990s was coupled with an attack on rentals, as the 1985 Boyer Decree removed rent control and tenancy protection for new rental contracts (see Observatorio DESC and PAH 2013). By the 1990s mortgages had become the most attractive means to access housing, particularly for low‐income households (López and Rodríguez 2010; Palomera 2014; Pareja Eastaway and San Marín Varo 2002). As a former Catalan Secretary of Housing told us in an interview: ‘[after the 1990s] the financial establishment hurled itself onto families, so that they would ask for [mortgage] credit’ (personal communication 7 May 2014).

The liberalisation of land laws (Roca Cladera and Burns 2000; Romero *et al*. 2012) accompanied the increased availability of mortgage credit, from €82.3 billion in 1992 to €934 billion in 2006 (Sánchez Martínez 2008). This led to an exponential growth in the construction sector and in real‐estate speculation (Charnock *et al*. 2014). Between 1997 and 2007, the provision of new housing stock rose to over 6 million, while house prices skyrocketed to over 200 per cent of their value in previous years (EMF 2011; García 2010; Naredo *et al*. 2008). Between 1997 and 2005, approximately 60 per cent of the total housing credit available in Spain financed the purchase of around 9 million housing units (Coq‐Huelva 2013; Naredo *et al*. 2008). The result was not only a stimulation of home ownership but also a significant boost to the economy (Banco de España 2003; Hoekstra *et al*. 2010).

The liberalisation of mortgage financing was embedded within broader European‐wide processes of market liberalisation (López and Rodríguez 2011). The adoption of the euro (1999) and the subsequent significant decrease in interest rates (from 16% in the early 1990s to 3% in 2004) contributed further to making Spain safe and attractive for international investors. Direct foreign investment in real estate increased by 102 per cent between 1998 and 2006 (García 2010), facilitating the process that enabled property titles to be traded as a financial asset (Harvey 1982) and turning land and housing into a form of fictitious capital (Christophers 2010). By 2007, 36 per cent of Spanish mortgage debt was securitised, with only the UK holding a higher percentage within the European Union at the time (Naredo *et al*. 2007). The number of housing units initiated increased from 500 000 units in 1999 to its peak of 865 561 in 2006 (EMF 2011), figures higher than housing production in the UK, France, Italy and Germany *combined* during the same period (López and Rodríguez 2011). By 2010, 82 per cent of the country's population qualified as homeowners, while social housing accounted for less than 2 per cent of the total housing stock. Already at this point, however, around 3.5 million new housing units remained unsold and empty, omens of an unfolding socioeconomic disaster (INE 2013).

## Financialising real estate, financialising life: mortgages as a ‘technology of power over life’ (1997–2007)

As changes in institutional and legislative frameworks fed the mortgage‐led building frenzy in Spain, housing supply kept rising alongside housing prices. But instead of causing alarm, this speculative carousel only animated the narrative of housing as a safe investment and fuelled the production of citizens as investors. From the late 1990s onwards, public administrations, real‐estate agencies, developers, builders and financial institutions alike chanted the same mantra: the price of housing never falls, housing is a safe investment (García Montalvo 2008). The ‘safe investment’ message was complemented by the mass media and bank advertisements suggesting a direct link between an elevated social status and home ownership (Palomera 2014, 3).

So long as interest rates were low and housing prices kept rising, the promotion of housing acquisition through mortgages as a safe investment was a winner. Financial institutions became increasingly aggressive and inventive in their outreach to clients, offering mortgages through the Internet, television ads and unsolicited mail (Fernández Rincón 2013). Becoming a homeowner appeared to be an easy affair, as citizens/customers were presented with tailor‐made mortgages to cater for their specific needs: ‘the young mortgage; the easy mortgage; the free mortgage; the open mortgage; the serene mortgage; the global mortgage; the paid off mortgage; the wild mortgage; the super mortgage; the revolution mortgage’ (Colau and Alemany 2012, 66–7).

Every single one of the mortgage‐affected people interviewed as part of this research highlighted the intense pressure they received during this period from a range of actors (state and banking institutions, the media, but also family and friends) to get a mortgage and buy a house. At the same time, the increasingly expensive rental market and the scarcity of social housing made buying a house appear to be the smartest option to ensure security and stability for one's self and family. Four informants underlined how the difficulty to meet increased rental prices and the inability to make rental deposits that ranged between €3000 and €6000 made them consider getting a mortgage. Many non‐Spanish informants also highlighted that, compared with renting, mortgaged home ownership promised more secure and stable housing, which to them was essential before they could bring their families from their country of origin. As S explained, her husband searched a long time for a rental flat before she arrived from Colombia but was unable to find one: ‘in the end he found mortgages, all mortgages, only mortgages’ (personal communication 8 September 2014). Several informants also underlined the ease with which they obtained their mortgages:

[in 2000] … I did not even have to go to a bank to ask for a mortgage. Everything was made easy for me. People came to you and offered [mortgages] with conditions that you never in your life thought you would be offered … They came to my house, to my workplace … If you went to an estate agent they would send a sales agent to your house, he ate you up, he told you many things, he described it in a lovely, wonderful way, that this was your life's dream, many things. You get excited, and you end up falling in their web. (Personal communication, N, 40‐year‐old male 11 June 2014)

While many heard that renting was ‘throwing money away’, half‐a‐dozen mortgage‐affected informants recounted the words of estate agents or bankers explaining why indebting oneself was the most sensible option:

rent a flat and you'll have to pay €800. Buy a flat and you'll pay €900. For €100 more, you live in your own flat and as time goes by that money is yours. (Personal communication 14 April 2014)

Under these conditions the number of mortgages issued rose from over 600 000 per annum in the early 2000s to over 1.3 million per annum in 2006 (Table 1). Between 2003 and 2006, financial institutions offered mortgages at 100 per cent, or even 120 per cent of the property's purchasing price and cross‐collateralised mortgages (particularly for immigrant households) with friends, relatives or acquaintances acting as guarantors or co‐owners on each other's mortgage. As a retired banker explained, these practices were mobilised to make figures appear properly tallied in the banks’ books:

The purchase‐sale price of a home was €100 000 but the mortgage granted was €130 000, and the Bank of Spain didn't meddle. How was this done? By making it look like there were four guarantors … It looked like they bought the flat together but there was no relationship or anything. Just to balance the numbers … (Personal communication 24 July 2014)

With financial institutions mobilising mortgages as a speculative form of rent, and land and housing titles given to ‘homeowners’ as claims on their future labour, Spain's new speculative cycle was further removed from real production than ever before. With 36 per cent of Spanish mortgage debt securitised in 2007 (Naredo *et al*. 2007) and the average ratio of loan to market price around 110 per cent (García Montalvo and Raya Vilchez 2012), mortgaged households were left vulnerable not only to the fluctuations of the local real‐estate market, but also to the fluctuations of interest rates and to the performance of unknown, unpredictable and complex financial dynamics.

## ‘Mortgaged lives’: The biopolitics of debt as fictitious wealth (1997–2007)

Ironically, while Spanish household debt was exponentially increasing, the boom in real‐estate prices produced a fictitious increase in recorded household wealth. Spanish household wealth appeared in official statistics to grow from 480 per cent of Gross Domestic Investment (GDI) in 1995 to 800 per cent of GDI in 2006 (Sánchez Martínez 2008). However, 540 per cent of the wealth recorded in 2006 corresponded to property wealth, which was calculated on the basis of inflated property values of largely mortgaged properties (Sánchez Martínez 2008). Owner‐occupiers were more than ever successfully produced as what Langley (2008) terms leveraged investors, that is, subjects for whom the shelter of their home would provide the key to future security and stability, as their investment was expected to grow and realise future returns. In the words of M, a mortgage‐affected woman:

As the price of flats went up [flats were presented to us] as if they were the best caviar in the world … But all that we, poor people, asked for was simply … a dignified home where we can live and work. (Personal communication 25 July 2014)

Like M, most mortgage holders did not see this offer of ‘caviar’ for the mirage that it was, and the supposed increase in household ‘wealth’ in effect corresponded to a real increase in household debt, which increased from €154.5 billion in 1999 to €674 billion in 2008 (EMF 2011). Household indebtedness rose from 55 per cent to 130 per cent of disposable income between 1997 and 2006 (Banco de España 2007). In December 2009, the European Central Bank recorded Spanish household debt at 84 per cent of annual Gross Domestic Product (GDP) (García 2010), a time when Spain was the country with the highest ratio of long‐term household mortgage debt to disposable income in the world (Naredo *et al*. 2007 2008).

The exponential increase of the indebtedness of Spanish households coincided with a significant decrease in wages (Table 2). The dismantling of traditional industrial activity, and the gradual reorientation of the Spanish economy towards the service sector after the 1980s were accompanied by changes in labour law that enabled workforce flexibilisation and permitted an increase in temporary and precarious contracts (TAIFA 2005). The construction sector played a key role in reorienting Spain's economy and flexibilising its labour force. Of the 8.1 million new jobs created between 1996 and 2007, 20 per cent were in the construction sector, and over 50 per cent were low‐skilled jobs in the service sector (Romero *et al*. 2012). During the same period, however, around 30 per cent of new contracts were for short‐term employment, and average salaries fell by 10 per cent in real terms, with 40 per cent of wage earners barely earning €1000 per month (López and Rodríguez 2010).

**Table 2:** Distribution of wage earners by income scale in Spain, 2007

Salary scale	0–€15 977	€15 977–€39 942	€39 942–€59 913	€59 913–€79 884	>€79 884
Number of wage earners	10 863 957	7 017 173	958 288	275 817	193 796
Foreigners (%)	16.7	4.6	1.9	2.4	4.2
Women (%)	50.4	35.4	28	22	14.2
% of total wage earners	59	35	4	1.2	0.8
% of total salaries	25.2	49.2	13	5.4	7.2
Average annual salary	**€**10 935	**€**26 175	**€**47 599	**€**68 111	**€**129 852

Despite a stark increase in labour precarity, at least one million mortgages were issued between 2003 and 2007 to vulnerable parts of the Spanish population (López and Rodríguez 2011). In 2007, when one third of work contracts were temporary and 60 per cent of the workforce lived on an average annual salary of €10 935, financial institutions were making mortgages available to an ever broader population base, thus making home ownership as precarious as job contracts (López and Rodríguez 2010, 233). Savings banks in particular enticed large segments of the population into entering high‐risk financial operations under the promise of, and belief in, an *ad infinitum* increase in the market value of their mortgage‐owned home.

Variable interest rates further increased the risk. During the second half of 2009, when Euribor rates had reached an unprecedented low (0.341% in September 2009),^5^ more than 80 per cent of mortgages in Spain were issued at variable interest rate, with over three quarters of these indexed to the 12‐month Euribor (EMF 2012). While variable interest rates had reached their historically lower point (to that date), Spain became the European country that issued the highest percentage of variable interest‐rate mortgages (Naredo *et al*. 2007, 84). This was one factor that gave birth to the concept of ‘Spanish‐style subprime’ (*subprime a la española*), which denoted the high risk incurred in these contracts (Calleja 2008; Colau and Alemany 2012; Naredo 2009). To top it all off, during the boom at least 30 000 mortgages were issued in foreign currencies (mainly Yen or Swiss Francs), apparently taking advantage of the then favourable currency exchange rates, a strategy that would backfire later as currency fluctuations made mortgage repayments up to 40 per cent more expensive (Quelart 2013).

## The mortgage or your life: facing the impossible choice of paying or being a debtor for life

Becoming heavily indebted in order to become a homeowner was something that ‘everyone was doing’ during the boom period. As one mortgage‐affected informant put it, getting a mortgage was as easy as ‘going out to buy bread’ (personal communication 4 September 2014). However, indebtedness started to rear its ugly head as unemployment started climbing from its historic low of under 9 per cent in 2006–2007 to over 26 per cent in 2013. As people became unemployed or experienced sharp salary decreases, they became unable to pay their mortgage debt. This was exacerbated in many cases by health problems, splitting up with a partner and/or increased monthly mortgage payments due to increases in interest rates or fluctuations in currency exchange rates. Two‐thirds of the mortgage‐affected informants underwent long periods of struggling, using all means possible to keep up with their mortgage repayments before they eventually faced no other choice than to stop paying altogether. As one interviewee underlined:

nobody simply stopped paying all of a sudden … Everyone will explain to you that they've done the same as I did; initially letting up on one or two mortgage payments, then trying again, asking for money, paying again for a while … (Personal communication 8 September 2014)

For the majority of our informants, part of the effort to keep up with mortgage repayments involved negotiating a solution with the bank. Over three quarters of our informants were offered either mortgage refinancing or a grace period of reduced monthly instalments for one or two years. Over 70 per cent of informants accepted these offers, but two out of eleven were unable to keep up with payments. Faced with the inability to put food on the table, and/or pay utility bills, they decided to stop paying. As F, a 30‐year‐old woman explained:

I did not want to stop paying, it's difficult for you to accept what is happening to you … I said to my husband: look, you are going to find work, I am going to keep going with the €800 I earn. We had to pay an instalment of €600 per month after refinancing, which is why my husband said: ‘but its €600, we're left with €200 to survive’ … Then came a moment when my husband said: ‘I don't know, I can no longer support your choice to keep paying the mortgage because we don't have any money to buy food. We either pay the mortgage or we stay alive’. (Personal communication 23 June 2014)

Ten of the eleven informants who either signed refinancing agreements or grace periods found themselves worse off, because after the end of these periods their monthly instalments increased significantly. Four informants who signed refinancing or grace periods noted that their banks did not explain or inform them about the implications. S explained that she and her husband were told by the bank that if they signed a grace period agreement ‘[the overall mortgage debt would not] increase; not even by one euro; you'll [just] pay less for one year [until] you find a job’ (personal communication 8 September 2014). At the point of negotiating a grace period, their outstanding balance was at €223 000. But when they read the terms and conditions in the small print of the new contract they noticed that at the end of the grace period their outstanding balance would increase by €11 200, to a total of €234 200. The impact of the increased indebtedness that refinancing or grace periods brought was amplified by the significant decrease in housing prices, falling up to 78 per cent^6^ in 2014 from their peak in 2008.

According to informants’ narratives, the punitive and disciplinary grip that mortgages held on their lives was revealed the moment they stopped paying their monthly instalments. Half of those interviewed recorded threats and hounding by the bank or debt collectors. R, a 35‐year‐old informant, explained:

When I stopped paying my mortgage, I went to inform the bank. [Their response was]: ‘No [you cannot do this]; you have to continue with payments no matter what; this is your fault, you shouldn't have gotten yourself into this. We're going to take away your flat, and on top of that, you'll still have to pay us.’ And I said: ‘hey, look, I've come for solutions, I already have problems’, but all they said was: ‘you need to pay, pay, pay’. I knew I couldn't pay the full instalment for that month [€1200]; the following month I sacrificed myself, and the month after that; just getting by, just getting by … (Personal communication 6 May 2014)

All informants explained how they fought long and hard to find solutions with their bank. On threats of foreclosure and eviction, some were offered loans at 10 per cent interest to pay back their mortgage debt with requirements including finding guarantors with salaries or fully paid properties as collateral. When they refused these deals, the bank responded that, according to Spanish law, their choice was to either pay up, or have their salary (above a base level of €967) and their goods confiscated until their debt was repaid. In many cases, the confiscation of salaries would have to last a full lifetime before repayment was completed.

The pressure on mortgage debt holders to pay instalments in full or lose their homes increased further after 2012 when, following a decade of promoting mortgage market deregulation, the European Central Bank subjected European banks to a series of stress tests that forced banks to improve their solvency with immediate effect. By this point, virtually all Spanish savings banks had been partially or fully rescued (nationalised) with Spanish and European funds and subsequently turned into banks or bought by existing banks. Most of these entities were heavily exposed to real‐estate debt, including defaulted mortgage contracts. Banks thus came under strong pressure to reclassify said debt as non‐performing and sell it off at a fraction of its nominal value to international distressed debt investors (Méndez and Pellicer 2013; see also Banco de España 2013; Brat and Bjork 2013). For example, in July 2014, 112 000 residential mortgages worth €6.4 billion were sold to the multinational investment and advisory firm Blackstone at almost half their nominal value (Munoz 2014).^7^ This practice casts families into unchartered waters and leaves them facing an even more precarious and uncertain future as there is often no office they can physically approach to renegotiate or pressure for a solution to their debt.

However, even before this massive sale of mortgage debt to international investors, banks were reluctant to engage in earnest with finding solutions for defaulting mortgage holders. According to mortgage‐affected people who were interviewed for this research, banks often used the fact that mortgages were securitised as an excuse to refuse debt forgiveness in exchange for repossessing homes, or to decline the provision of a social rental contract to defaulting families (i.e. allow defaulted mortgage holders to stay in their repossessed homes at a rent no higher than 30 per cent of their household's income). Given the complexity of the processes involved and the lack of reliable and accessible information for the general public, many mortgage‐affected people did not know whether their mortgage debt was securitised or sold‐off (or both, one after the other). Indeed, very few people understood in depth the difference between the two processes, and what it meant with respect to their own and the banks’ rights and obligations. One male mortgage‐affected informant described securitisation as a process that turned people's lives essentially into numbers, that were then sold to financial brokers to speculate with. Once a family is unable to pay, the broker in effect ‘manages the portfolio classified as “junk”. So what does that make us? Rubbish. Scum’ (personal communication 15 May 2014). Whether referring to securitisation or sell‐off, this statement exemplifies how mortgage defaulters/citizens are produced as financial objects of speculation in order to expand circuits of capital accumulation, and are subsequently ‘used up and discarded’ (Dean 2012) as their lives and current and future labour become the subject of speculative financial practices.

## Facing evictions: face to face with the violence of financial capitalism

The crumble of the so‐called ‘Spanish miracle’ post‐2007 and the set in of the economic crisis made even more stark the role of mortgages as a ‘secondary form of exploitation’ (Harvey 1982, 285). The role of mortgages as a disciplinary tool was exposed in a dramatic manner when, as many mortgage holders became unable to service their debt, an increasing number of homes were foreclosed – 570 000 between 2008 and 2014 inclusive, according to Spain's General Council of Judicial Powers – and hundreds of thousands of families were forcefully evicted from their mortgaged homes. It is estimated that at least 250 000 mortgaged families were evicted between 2008 and 2014 (Cano Fuentes *et al*. 2013).^8^ The official figure of 163 000 evictions of mortgaged homes between 2008 and 2011 only records repossession orders issued by Senior Courts (*Tribunal Superior de Justicia*), while repossession orders issued by County Courts (*Juzgados de primera instancia e instrucción*) during the same period remain unrecorded (Cano Fuentes *et al*. 2013).

The everyday impact of what Marazzi (2007) terms ‘the violence of financial capitalism’ manifested in evictions becomes even more arresting when one also adds the fact that, due to recent collapse in housing prices, evicted households in Spain remain liable for repaying their outstanding mortgage debt even after their homes are repossessed. Current mortgage legislation in Spain dictates that a private insolvent debtor cannot cancel their debt even after the repossession and sale of their asset by their creditor if the sale of the asset fails to cover in full the outstanding debt plus interest on late repayments and administrative or legal costs (Castillo 2013). As many informants noted, the total of outstanding debt plus interest and legal and administrative fees after repossession (and) eviction can easily add up to €100 000–€300 000 depending on the outstanding balance, the original purchasing price, and the decrease in housing prices since the original purchase. So, as soon as liquidity dried up, unemployment increased and housing prices decreased, many mortgaged homeowners unwittingly became either homeless proletariats, evicted yet still indebted for life to their creditors, or indebted proletarianised homeowners who were nevertheless ‘in material and housing‐class terms, barely distinguishable from renters … simply paying rent to the new landlord’ (Wyly *et al*. 2009, 338), who now exercise new forms of financial violence. As expressed by a 65‐year‐old male former entrepreneur:

I consider myself from a class that no longer exists, that has been wiped off the map. Middle. There is no middle class anymore; now, we are nothing. (Personal communication 30 May 2014)

The above material exemplifies how mortgages contributed to the creation of the new biopolitical subject, that Lazzarato (2012) terms the indebted person, whose life unwittingly becomes dependent on the performance of global financial markets. Suddenly, the fluctuation of real‐estate prices, Euribor rates or currency exchange values became factors determining not only households’ access to housing, food and a decent living (Benito 2007; Crouch 2009; Doling and Elsinga 2013; Lowe *et al*. 2012), but also levels of self‐esteem and belonging to a family or a community. Indeed, when unable to pay, all mortgage‐affected informants were riddled with deep‐seated feelings of guilt, fear and even shame, often exacerbating a range of family and personal health problems. Many experienced depression, suicidal thoughts or suicide attempts, and saw themselves as outcast from family or social life (also see Observatorio DESC and PAH 2013, 113–15). C, a female informant, explained her despair when her father‐in‐law said to her and her husband: ‘it is shameful that you find yourselves in this situation’ (personal communication 15 July 2014).

For the people who Ada Colau describes as living in a contemporary regime of slavery in the opening quote of this article, dignity, welfare, self‐esteem, family/community belonging, health and well‐being have become heavily dependent on their ability to fulfil their mortgage repayment obligations under increasingly volatile market and labour conditions. It is a situation where an unscrupulous manipulation of lives and bodies lies at the heart of economic and political strategies and decision‐making (Rose 2007). M underlines this:

We shall continue being your slaves; but at least don't take away our housing, let us breathe a bit. We are their slaves, waiting for them to toss us something. Like a dog, when you bring it food it gets happy. When you have work you are happy because you can pay, you can live, but if you have a debt and you are a defaulter, you can't even buy a second hand washing machine. (Personal communication 25 July 2014)

Many informants also reflected on how attending actions to block evictions made them realise how their own labour and life are also bound to servicing debt. Witnessing the bank evict a woman from her flat, F remarked that they were: ‘taking away the woman's working years, taking away all her life’ (personal communication 23 June 2014). S, another female informant, stressed how impotent she felt when facing an eviction: ‘the bank sees the person as if they were money, they don't see the human side. Once they suck you dry they are no longer interested nor do they care about how you suffer’ (personal communication 17 July 2014). For many, mortgage defaults and evictions became a form of the ‘invasion of the real’ (Rancière 2004): the realisation that they had never really been homeowners or middle class, just a proletariat indebted for life to their creditors. R noted:

you realised, oh, this is what speculation is … it made me think that yes there are rich people … and poor people. There are no people in the middle or anything. (Personal communication 6 May 2014)

C also describes the moment she realised what mortgages really meant:

Thanks to finding myself in this situation I saw reality for what it is. Before, I used to live my life earning a good salary … I didn't realise that people had a really tough time. You know what I mean? So although it doesn't make me happy, it has opened my eyes a bit. (Personal communication 15 July 2014)

## Conclusion

The paper conceptualised mortgages as a biopolitical technology and unpacked mortgage debt as a globally fuelled but locally embedded and socially embodied process that forges an intimate connection between everyday life, global practices of financial speculation and the creation of urban futures. By expanding the analysis to the largely overlooked embodied practices related to signing mortgage contracts and servicing mortgage debt, we contend that these understudied embodied practices present a ‘missing link’ in our analysis of housing financialisation and the related debt crisis.

Our analysis undercuts current debates in political economy, and adds a new dimension to the debates on financialisation and subjectification by showing that the production of mortgaged subjects as leveraged investors is at least as crucial as macroeconomic and institutional changes in expanding the dynamics of urban capital accumulation under a financialised world economy. Focusing on the ongoing mortgage and evictions crisis in Spain, we showed how the financialisation of housing was achieved not only through the liberalisation of economic and institutional practices, but also through the production of citizens as real‐estate investors, connecting them into speculative complex financial relations with the potential to severely disrupt and dismantle their livelihood. The frenzy to become an investor‐homeowner through easy access to mortgage credit subjected the productive and reproductive capacity of livelihoods to fuel an ever‐expanding cycle of real‐estate speculation and capital accumulation. The issuing of about one million mortgages per year in Spain between 2003 and 2007, a period during which real wages fell, exposed a large proportion of the population to the rent‐extracting mechanisms of an increasingly volatile financial sector. In return for the promise of home ownership, mortgage contracts became the mechanism that turned interest rates and real‐estate prices into factors determining not only people's access to housing, but also their sense of belonging and self‐esteem, their capacity to care for their elderly or their children, and so forth.

We have thus illustrated how, from the mid‐1990s to the late 2000s, mortgages performed three significant and mutually intertwined functions. First, they stimulated the construction sector, fuelling real‐estate speculation and capital expansion in the wake of economic restructuring. Second, they commodified the indebtedness of the labour force, as debt claims over the current and future labour of the work force became packaged as financial products to be traded at global financial markets through special‐purpose vehicles and securitisation practices. Third, mortgages served as a disciplinary mechanism for a precarious labour force that found themselves under conditions of increased volatility after having signed mortgaged contracts. As Spanish banks and global financial institutions became rent extractors, a new form of accumulation came into play producing a new biopolitical subject: the indebted person. The documentation of these three intertwined roles that mortgages played since the 1990s in Spain support our claim that mortgages operated as a biopolitical technology that engineered an intimate relationship between everyday life and financial institutions.

The conceptual framework we develop here advocates further historical and geographical analysis of the biopolitics of mortgaged home ownership. It calls for further attention to the overlooked yet central role that embodied practices of debt play in enabling the flow of capital to be channelled in and through land in order to enact urban transformation not only in the contemporary context, but historically too. A better understanding of the biopolitics of debt can also enable us to better contextualise the increasing importance of resistance strategies and struggles that strive to disentangle the web of life from its direct dependency on capital flows. As millions of people across the world are affected by mortgage defaults and evictions (Roy 2010; Rolnik 2013), this is a process akin to a social disaster that requires urgent attention, systematic documentation and fresh conceptualisation.

## Notes

2 Although a comprehensive critique of the concept of ‘housing financialisation’ lies outside the scope of this paper (for a critical review see Aalbers 2008; Christophers 2015), we should note for clarity that the term ‘housing financialisation’ is used throughout the paper to depict the global expansion of speculative real estate investment through financial markets as a means to (temporarily) overcome the fundamental contradiction in the secondary circuit of capital (Aalbers 2008; Gotham 2006; Harvey 1978).

3 According to Spain's General Council of Judicial Power (personal communication, February 2016), 43 000 foreclosures (which generally equate to mortgage evictions) were recorded in 1995, while in 2014 this number soared to 133 000. According to the same source, a systematic data collection on foreclosures and evictions is only available from 1995 onwards, as data‐gathering methods were different in previous years.

4 SPVs operate as a ‘subsidiary company with an asset/liability structure and legal status that makes its obligations secure even if the parent company goes bankrupt’ (http://www.investopedia.com/terms/s/spv.asp).

5 A full list of Euribor rates by month and year is available at http://www.euribor-rates.eu/euribor

6 This was the highest fall in the market value of a single property reported among our informants: from €275 000 in 2007 to €60 000 in 2013.

7 These loans were formerly held by Cataluyna Caixa, a nationalised bank formed from three ‘rescued’ savings banks.

8 The fact that there are no reliable official figures for the exact number of foreclosures and mortgage evictions is now recognised by the government as a serious issue, and is being addressed through the mandate to the National Statistics Institute to prepare official eviction statistics (Díaz Guijarro 2013).
